# Molecular Epidemiology of OXA-48 and NDM-1 Producing *Enterobacterales* Species at a University Hospital in Tehran, Iran, Between 2015 and 2016

**DOI:** 10.3389/fmicb.2020.00936

**Published:** 2020-05-28

**Authors:** Hamid Solgi, Shoeib Nematzadeh, Christian G. Giske, Farzad Badmasti, Fredrik Westerlund, Yii-Lih Lin, Gaurav Goyal, Vajihe Sadat Nikbin, Amir Hesam Nemati, Fereshteh Shahcheraghi

**Affiliations:** ^1^Division of Clinical Microbiology, Department of Laboratory Medicine, Amin Hospital, Isfahan University of Medical Sciences, Isfahan, Iran; ^2^Division of Clinical Microbiology, Department of Laboratory Medicine, Karolinska Institutet, Karolinska University Hospital, Stockholm, Sweden; ^3^Department of Bacteriology, Pasteur Institute of Iran, Tehran, Iran; ^4^Division of Chemical Biology, Department of Biology and Biological Engineering, Chalmers University of Technology, Gothenburg, Sweden

**Keywords:** carbapenemase-producing *Enterobacterales*, PFGE, MLST, ST147, optical DNA mapping

## Abstract

Carbapenem-resistant *Enterobacterales* (CRE) is an increasing problem worldwide. Here, we examined the clonal relatedness of 71 non-repetitive CRE isolates collected in a university hospital in Tehran, Iran, between February 2015 and March 2016. Pulsed-field gel electrophoresis (PFGE) and MLST were used for epidemiological analysis. Screening for antibiotic resistance genes, PCR-based replicon typing, conjugation experiments, and optical DNA mapping were also performed. Among all 71 isolates, 47 isolates of *Klebsiella pneumoniae* (66.2%), eight *Escherichia coli* (11.2%), five *Serratia marcescens* (7%), and two *Enterobacter cloacae* (2.8%) harbored *bla*_NDM–1_ and *bla*_OXA–48_ genes together or alone. PFGE analysis revealed that most of the OXA-48- and NDM-1-producing *K. pneumoniae* and all of OXA-48-producing *S. marcescens* were clonally related, while all eight *E. coli* and two *E*. *cloacae* isolates were clonally unrelated. The predominant clones of carbapenemase-producing *K. pneumoniae* associated with outbreaks within the hospital were ST147 (*n* = 13) and ST893 (*n* = 10). Plasmids carrying *bla*_NDM–1_ and *bla*_OXA–48_ were successfully transferred to an *E. coli* K12-recipient strain. The *bla*_OXA–48_ gene was located on an IncL/M conjugative plasmid, while the *bla*_NDM–1_ gene was located on both IncFII ∼86-kb to ∼140-kb and IncA/C conjugative plasmids. Our findings provide novel epidemiologic data on carbapenemase-producing *Enterobacterales* (CPE) in Iran and highlight the importance of horizontal gene transfer in the dissemination of *bla*_NDM–1_ and *bla*_OXA–48_ genes. The occurrence and transmission of distinct *K. pneumoniae* clones call for improved infection control to prevent further spread of these pathogens in Iran.

## Introduction

Carbapenems are broad-spectrum beta-lactam agents that are frequently used as a last resort to treat serious infections caused by multidrug-resistant *Enterobacterales*. Resistance to carbapenems mainly depends on the production of carbapenemase enzymes. Carbapenemase-producing *Enterobacterales* (CPE) are increasingly reported and represent a major public health threat (Tängdén and Giske, 2015). The most clinically significant carbapenemases in *Enterobacterales* include the class A (KPC type), class B (metallo-β-lactamases [MBLs] [i.e., VIM, IMP, and NDM types]), and class D carbapenem-hydrolyzing β-lactamases (OXA-48-like enzymes) (Nordmann et al., 2011; Tängdén and Giske, 2015). NDM-1 and OXA-48 β-lactamases were initially identified in India and Turkey, respectively, and then spread to various countries worldwide including India, the Middle East, and Mediterranean countries (Yong et al., 2009; Johnson and Woodford, 2013; Sartor et al., 2014; Jamal et al., 2016; Solgi et al., 2017b). There is a lot of pilgrimage tourism and business travel between Iran and neighboring countries such as Iraq, Afghanistan, Pakistan, Turkey, and the Persian Gulf, so travelers with CPE colonization may be the vectors for spread of resistant strains. In the scope of outbreaks in Iran, diverse sequence types (STs) of dominant OXA-48- and NDM-producing *Klebsiella pneumoniae* have been identified in outbreaks or solitary case reports (STs 11, 893, 147, and 915) (Solgi et al., 2017a, 2018). VIM-2-producing *K. pneumoniae* ST23 has been reported in Iran more recently (Mohammad Ali Tabrizi et al., 2018).

The dissemination of OXA-48 and NDM-1 among *Enterobacterales* is mediated by the rapid spread of broad host-range conjugative plasmids. The *bla*_NDM–1_ gene has been detected on plasmids of various incompatibility groups: IncF, IncA/C, IncL/M, IncH, IncN, and IncX3 or untypeable (Voulgari et al., 2014). The *bla*_OXA–48_ gene has also been carried by various plasmids types including IncL/M, IncN, and IncA/C (Guo et al., 2016). Up until today, only one study has reported the finding of the prevalence and distribution of carbapenem resistance among *Enterobacterales* isolates in Iran (Shahcheraghi et al., 2017). However, limited data about the sequence type of CRE isolates that has spread in Iran were available.

Here, we investigated the prevalence of ESBL and carbapenemase genes, to explore the distribution of plasmid replicons, and molecular epidemiology of CPE isolated in an Iranian hospital.

## Materials and Methods

### Bacterial Strains

In this cross-sectional study, a total of 71 non-repetitive carbapenem-resistant *Enterobacterales* (CRE) clinical isolates resistant to at least one of the carbapenems (imipenem, meropenem, or ertapenem) were collected at the Loghman Hakim Educational Hospital, a 496-bed university hospital in Tehran (Iran) between February 2015 and March 2016. All isolates were identified by standard biochemical tests and API 20E (bioMérieux, Marcy-l’Etoile, France).

### Antimicrobial Susceptibility Testing and Phenotypic Assay

Antimicrobial susceptibility testing of 10 antibiotics (imipenem, meropenem, ertapenem, cefepime, cefotaxime, ceftazidime, aztreonam, amikacin, gentamicin, and ciprofloxacin) was done by a standard disk diffusion method according to the Clinical and Laboratory Standards Institute [CLSI] (2017) guidelines. The minimal inhibitory concentration (MIC) determinations for carbapenems (imipenem, meropenem, and ertapenem) were performed by gradient test strips (Liofilchem, Italy) based on Clinical and Laboratory Standards Institute [CLSI] (2017) guidelines. MICs of colistin were determined by broth macrodilution method using colistin sulfate (Sigma-Aldrich), and EUCAST breakpoints were used for interpretation (EUCAST, 2017). *Escherichia coli* ATCC 25922 was used as quality control. Initial screening for the presence of carbapenemases was done by the modified Hodge test (MHT) test by following the Clinical and Laboratory Standards Institute [CLSI] (2017) guideline.

### Molecular Detection of Genes Encoding Carbapenemases and ESBLs

Plasmid DNA was extracted using the Gene JET Plasmid Maxi-Prep Kit (Thermo Scientific). The presence of genes encoding carbapenemases (*bla*_KPC_, *bla*_GES_, *bla*_VIM_, *bla*_IMP_, *bla*_NDM_, and *bla*_OXA–48_) and extended-spectrum β-lactamases (ESBL) (*bla*_CTX–M_) and further beta-lactamases (*bla*_TEM_, *bla*_SHV_) were detected by PCR amplification using specific primers as described previously (Poirel et al., 2011; Shahcheraghi et al., 2013), followed by sequencing (Macrogen Research, Seoul, South Korea).

### Molecular Typing

The genetic relatedness of CPE isolates was investigated by pulsed-field gel electrophoresis (PFGE). The genomic DNA of the CPE isolates and reference marker *Salmonella* serotype Braenderup strain H9812 were digested by *Xba*I endonuclease, which was performed with a CHEF-DRIII system (Bio-Rad Laboratories) as previously described (Tenover et al., 1995). A similarity coefficient was obtained using Dice coefficients. Cluster analysis was done with the unweighted pair group method with arithmetic averages (UPGMA). Isolates that exhibited similarity cut-off ≥80% of their banding patterns were considered to belong to the same clonal lineage (pulsotypes). Multilocus sequence typing (MLST) was performed according to the protocol described on the Pasteur Institute MLST website^1^ for *K. pneumoniae*, MLST website for *E. coli*^2^, and MLST website for *Enterobacter cloacae*^3^.

### Conjugation Experiments and PCR-Based Replicon Typing

Conjugation experiments were done using the *bla*_NDM–1_ and *bla*_OXA–48_ producers as the donors and *E. coli* K12 [F^–^ lac ^+^ Nal (r)] as the recipient strain (Filter mating). Isolates LO35, LO89, LO112, LO179, LO271, and LO273 which harbored only the *bla*_NDM–1_ or *bla*_OXA–48_ gene, and isolates LO149, LO155, and LO204, which harbored the *bla*_OXA–48_ and *bla*_NDM–1_ genes, were selected and used. Transconjugants were selected on a MacConkey agar plate containing 32 mg/L nalidixic acid (Sigma-Aldrich) and 1 mg/L MEM (MAST, Merseyside, United Kingdom) (Lyimo et al., 2016) and were confirmed to have *bla*_NDM–1_ and *bla*_OXA–48_ by PCR analysis. PCR-based replicon typing analysis (PBRT) was performed to determine the plasmid incompatibility (Inc) groups for all CPE strains and the obtained transconjugants (Carattoli et al., 2005).

### Plasmid Extraction

Plasmid DNA was prepared from an overnight culture with NucleoBond^®^ Xtra Midi kit for isolates according to the manufacturer’s description for high-copy plasmid purification (Müller et al., 2016a). Eluted plasmid DNA is then precipitated with isopropanol and washed with 70%; the dried pellet was reconstituted TE buffer, pH 8.0. The DNA concentration and purity were determined using the Qubit 3.0 Fluorometer.

### Optical DNA Mapping in Nanochannels for Plasmid Analysis

The presence of the *bla*_NDM–1_ gene on plasmids from isolates LO94, LO204, LO271, LO247, LO64, LO63, LO89, and LO149 was investigated using optical DNA mapping (Müller and Westerlund, 2017). For this, Cas9 enzyme (PNA Bio Inc., Newbury Park, CA, United States) was used to make a site-specific cut at the *bla*_NDM–1_ gene (target gene sequence was 5′-CGGTATGGACGCGCTGCATG-3′, RNA was synthesized by Dharmacon Inc., Lafayette, CO, United States) on the plasmids (Müller et al., 2016b). Cas9 will cut all the *bla*_NDM–1_ gene-carrying plasmids in each isolate at the same location which would show as a consensus cut site in the ODM data. For the plasmids not carrying the *bla*_NDM–1_ gene, we expected randomly distributed cuts.

After the Cas9 reaction, the plasmids were stained using YOYO-1 and Netropsin which created an emission intensity pattern along the DNA, with dark AT-rich regions and bright GC-rich regions (Nyberg et al., 2012; Nilsson et al., 2014). Netropsin prevents the binding of the fluorescent YOYO-1 to AT-rich regions which results in the formation of a variation in intensity, a DNA barcode. Plasmids were stretched to their full contour lengths by confining them in 100 × 150-nm^2^ nanofluidic channels and imaged using an EMCCD camera. For each of the eight isolates, hundreds of plasmids were imaged and analyzed. The barcodes were aligned, clustered based on similarity, and compared among the isolates using custom-built MATLAB routines (Müller et al., 2016a). Lambda phage DNA was used as an internal control to correlate the length in pixels with the length in base pairs, and this correlation factor was then used to estimate plasmid sizes.

## Results

### Bacterial Isolates

During the study period, 71 clinical CRE isolates were collected from 44 male and 27 female patients. These isolates mainly belonged to the species *K. pneumoniae* (56/71, 78.8%), *E. coli* (8/71, 11.2%), *Serratia marcescens* (5/71, 7%), and *E. cloacae* (2/71, 2.8%). Twenty-two isolates (31%) were isolated from an ICU poisoning ward, whereas the remaining of isolates were recovered from other ward. The majority of the isolates were from urine (30/71, 42.2%) and tracheal (24/71, 33.8%) specimens. Other sample types included blood (6/71; 8.4%), wound secretions (5/71; 7%), sputum (3/71; 4.2%), catheter (2/71; 2.8%), and cerebrospinal fluid (1/71; 1.4%).

### Antimicrobial Susceptibility

Susceptibility profiles against ten antimicrobials agents are listed in Table 1. As expected, the majority of the CRE isolates exhibited resistance to most β-lactams. Most of the isolates were also resistant to ciprofloxacin (70/71 98.6%) and gentamicin (42/71 59.1%). On the other hand, most of them were susceptible to amikacin (46/71 64.8%), and all isolates were susceptible to colistin, with MICs ≤ 1 mg/L. Based on phenotypic detection, 40 out of the 62 isolates (64.5%) were positive for MHT.

**TABLE 1:** Clinical information and molecular characteristics of 71 carbapenem-resistant *Enterobacterales* isolated from a university hospital in Tehran, Iran.

Patient/Strain	Species	Carbapenemase genes	Associated β-lactamases	Inc group^a^	ST	Specimen	Hospitalization unit	Resistance phenotype	MIC (μ g/ml)		
									
									ERT	MEM	IPM
**LO-1**	*K. pneumoniae*	NDM-1, OXA-48	CTX-M-15, TEM-1, SHV-106	IncFII, IncL/M	ST15	Tracheal	Emergency ICU	CAZ, CTX, FEP, CIP	8	8	8
**LO-7**	*K. pneumoniae*	NDM-1	CTX-M-15, TEM-1, SHV-199	IncFII	ST893	Tracheal	Poisoning ICU	CAZ, CTX, FEP, GEN, CIP	8	8	8
**LO-8**	*K. pneumoniae*	–	CTX-M-15, TEM-1	ND	ND	Wound	Poisoning ICU	CAZ, CTX, FEP, CIP	4	1	>4
**LO-17**	*K. pneumoniae*	NDM-1	CTX-M-15, SHV-1	IncFII	ND	Urine	Nerves of men	CAZ, CTX, FEP, AMK, GEN, CIP	8	32	64
**LO-20**	*K. pneumoniae*	–	CTX-M-15, TEM-1, SHV-1	ND	ND	Tracheal	Poisoning ICU	CAZ, CTX, FEP, CIP	4	0/5	>4
**LO-21**	*K. pneumoniae*	–	CTX-M-15, TEM-1, SHV-1	ND	ND	Tracheal	Poisoning ICU	CAZ, CTX, FEP, CIP	2	1	>4
**LO-30**	*K. pneumoniae*	–	CTX-M-15, TEM-1, SHV-1	ND	ND	Urine	Poisoning ICU	CAZ, CTX, FEP, CIP	4	0/5	>4
**LO-36**	*K. pneumoniae*	NDM-1	CTX-M-15, TEM-1, SHV-1	IncFII	ND	Urine	Poisoning ICU	CAZ, CTX, FEP, GEN, CIP	8	4	>4
**LO-56**	*K. pneumoniae*	–	CTX-M-15, TEM-1	ND	ND	Wound	Surgery	CAZ, CTX, FEP, CIP	2	1	>4
**LO-63**	*K. pneumoniae*	NDM-1	CTX-M-15, TEM-1, SHV-11	UT	ST147	Wound	Surgery	CAZ, CTX, FEP, AMK, GEN, CIP	8	32	32
**LO-64**	*K. pneumoniae*	NDM-1, OXA-48	CTX-M-15, TEM-1, SHV-199	IncL/M	ST893	Tracheal	Poisoning ICU	CAZ, CTX, FEP, CIP	8	32	64
**LO-68**	*K. pneumoniae*	NDM-1	CTX-M-15, TEM-1, SHV-11	IncFII	ST147	Sputum	Infectious	CAZ, CTX, FEP, AMK, GEN, CIP	8	32	256
**LO-70**	*K. pneumoniae*	NDM-1	CTX-M-15, TEM-1, SHV-1	IncFII	ND	Tracheal	General ICU	CAZ, CTX, FEP, CIP	8	8	8
**LO-77**	*K. pneumoniae*	NDM-1	CTX-M-15, TEM-1, SHV-11	IncFII	ST147	Tracheal	General ICU	CAZ, CTX, FEP, AMK, GEN, CIP	8	8	32
**LO-78**	*K. pneumoniae*	NDM-1	CTX-M-15, TEM-1, SHV-12	UT	ST147	Tracheal	Emergency ICU	CAZ, CTX, FEP, AMK, GEN, CIP	8	32	32
**LO-80**	*K. pneumoniae*	NDM-1	CTX-M-15, TEM-1, SHV-11	IncFII	ST147	Urine	Infectious	CAZ, CTX, FEP, AMK, GEN, CIP	8	32	64
**LO-82**	*K. pneumoniae*	NDM-1, OXA-48	CTX-M-15, TEM-1, SHV-199	IncL/M	ST893	Urine	Internal emergency	CAZ, CTX, FEP, AM, GEN, CIP	8	32	256
**LO-88**	*K. pneumoniae*	OXA-48	CTX-M-15, TEM-1, SHV-1	IncL/M	ND	Urine	Internal emergency	CAZ, CTX, FEP, GEN, CIP	8	4	4
**LO-89**	*K. pneumoniae*	NDM-1	CTX-M-15, TEM-1, SHV-11	IncFII	ST147	Urine	Outpatient	CAZ, CTX, FEP, AMK, GEN, CIP	8	16	32
**LO-91**	*K. pneumoniae*	OXA-48	CTX-M-15, TEM-1, SHV-1	IncL/M	ST377	Tracheal	Emergency ICU	CAZ, CTX, FEP, AM, GEN, CIP	8	8	8
**LO-94**	*K. pneumoniae*	NDM-1	CTX-M-15, SHV-199	UT	ST16	Tracheal	Infectious	CAZ, CTX, FEP, GEN, CIP	8	4	4
**LO-95**	*K. pneumoniae*	OXA-48	CTX-M-15, TEM-1, SHV-199	IncL/M	ST893	Tracheal	Internal emergency	CAZ, CTX, FEP, GEN, CIP	8	2	>4
**LO-97**	*K. pneumoniae*	OXA-48	CTX-M-15, TEM-1, SHV	IncL/M	ST16	Urine	Emergency ICU	CAZ, CTX, FEP, AMK, GEN, CIP	8	2	>4
**LO-106**	*K. pneumoniae*	NDM-1	CTX-M-15, TEM-1, SHV-1	IncFII	ND	Blood	Poisoning ICU	CAZ, CTX, FEP, AMK, GEN, CIP	4	4	2
**LO-110**	*K. pneumoniae*	OXA-48	CTX-M-15, TEM-1, SHV-199	IncL/M	ST893	Tracheal	Poisoning ICU	CAZ, CTX, FEP, CIP	4	2	>4
**LO-114**	*K. pneumoniae*	NDM-1	CTX-M-15, SHV-1	IncFII	ST657	Tracheal	Internal emergency	CAZ, CTX, FEP, CIP	8	8	8
**LO-119**	*K. pneumoniae*	–	CTX-M-15, TEM-1, SHV-1	ND	ND	Urine	Emergency ICU	CAZ, CTX, FEP, GEN, CIP	2	1	>4
**LO-121**	*K. pneumoniae*	OXA-48	TEM-1, SHV-199	IncL/M	ST893	Sputum	Internal emergency	CAZ, CTX, FEP, CIP	8	8	8
**LO-123**	*K. pneumoniae*	NDM-1	CTX-M-15, SHV-1	IncFII	ST35	Tracheal	Poisoning ICU	CAZ, CTX, FEP, AMK, GEN, CIP	8	32	32
**LO-125**	*K. pneumoniae*	OXA-48	CTX-M-15, TEM-1, SHV-182	IncL/M	ST11	Urine	Internal emergency	CAZ, CTX, FEP, CIP	4	2	>4
**LO-126**	*K. pneumoniae*	OXA-48	CTX-M-15, TEM-1, SHV-199	IncL/M	ST893	Urine	Poisoning ICU	CAZ, CTX, FEP, CIP	4	1	>4
**LO-147**	*K. pneumoniae*	NDM-1, OXA-48	CTX-M-15, TEM-1, SHV-106	IncFII, IncL/M	ST15	Tracheal	General ICU	CAZ, CTX, FEP, AMK, GEN, CIP	8	32	32
**LO-149**	*K. pneumoniae*	NDM-1, OXA-48	CTX-M-15, TEM-1, SHV-106	IncFII, IncL/M	ST15	Tracheal	Poisoning ICU	CAZ, CTX, FEP, AMK, GEN, CIP	8	32	32
**LO-154**	*K. pneumoniae*	NDM-1	CTX-M-15, TEM-1, SHV-199	UT	ST16	Urine	Internal emergency	CAZ, CTX, FEP, AMK, GEN, CIP	8	32	32
**LO-155**	*K. pneumoniae*	NDM-1, OXA-48	CTX-M-15, TEM-1, SHV-199	IncL/M	ST893	Tracheal	Poisoning ICU	CAZ, CTX, FEP, CIP	8	32	32
**LO-179**	*K. pneumoniae*	NDM-1	CTX-M-15, TEM-1, SHV-199	IncFII	ST16	Urine	Infectious	CAZ, CTX, FEP, AMK, GEN, CIP	8	32	32
**LO-181**	*K. pneumoniae*	NDM-1	CTX-M-15, TEM-1, SHV-1	IncFII	ST147	Urine	Surgery	CAZ, CTX, FEP, AMK, GEN, CIP	8	16	8
**LO-191**	*K. pneumoniae*	NDM-1	–	IncFII	ST1308	Wound	Surgery	CAZ, CTX, FEP	8	4	4
**LO-204**	*K. pneumoniae*	NDM-1, OXA-48	CTX-M-15, TEM-1, SHV-106	IncFII, IncL/M	ST15	Catheter	Surgery	CAZ, CTX, FEP, AMK, GEN, CIP	8	32	256
**LO-216**	*K. pneumoniae*	–	CTX-M-15, SHV-199	ND	ND	Urine	Internal emergency	CAZ, CTX, FEP, CIP	2	0/5	>4
**LO-217**	*K. pneumoniae*	NDM-1	CTX-M-15, SHV-199	IncFII	ST16	Urine	General ICU	CAZ, CTX, FEP, GEN, CIP	8	16	24
**LO-246**	*K. pneumoniae*	–	CTX-M-15, TEM-1, SHV-1	ND	ND	Tracheal	Poisoning ICU	CAZ, CTX, FEP, AMK, GEN, CIP	4	2	>4
**LO-247**	*K. pneumoniae*	NDM-1, OXA-48	CTX-M-15, TEM-1, SHV-199	IncFII	ND	Catheter	Neurosurgery	CAZ, CTX, FEP, CIP	8	8	4
**LO-251**	*K. pneumoniae*	NDM-1, OXA-48	CTX-M-15, TEM-1, SHV-199	IncL/M	ST893	Tracheal	Poisoning ICU	CAZ, CTX, FEP, CIP	8	4	2
**LO-261**	*K. pneumoniae*	OXA-48	TEM-1, SHV-199	IncL/M	ST893	Tracheal	Infectious	CAZ, CTX, FEP, CIP	8	4	4
**LO-262**	*K. pneumoniae*	NDM-1	TEM-1, SHV-172	IncFII	ST147	Urine	Neurosurgery	CAZ, CTX, FEP, AMK, GEN, CIP	8	32	256
**LO-263**	*K. pneumoniae*	NDM-1, OXA-48	TEM-1, SHV-12	IncL/M	ST147	Urine	Neurosurgery	CAZ, CTX, FEP, AMK, GEN, CIP	8	32	128
**LO-264**	*K. pneumoniae*	–	CTX-M-15, TEM-1, SHV-1	ND	ND	Tracheal	Poisoning ICU	CAZ, CTX, FEP, GEN, CIP	4	ND	ND
**LO-268**	*K. pneumoniae*	OXA-48	TEM-1	IncL/M	ST23	Sputum	Neurosurgery	CAZ, CTX, FEP, GEN, CIP	8	4	4
**LO-269**	*K. pneumoniae*	NDM-1	CTX-M-15, TEM-1, SHV-11	IncFII	ST147	Urine	Internal emergency	CAZ, CTX, FEP, AMK, GEN, CIP	8	16	4
**LO-270**	*K. pneumoniae*	NDM-1	CTX-M-15, TEM-1, SHV-11	IncFII	ST147	Urine	Infectious	CAZ, CTX, FEP, AMK, GEN, CIP	8	16	4
**LO-271**	*K. pneumoniae*	NDM-1	CTX-M-15, TEM-1, SHV-12	IncFII	ST147	Blood	Infectious	CAZ, CTX, FEP, AMK, GEN, CIP	8	16	32
**LO-272**	*K. pneumoniae*	NDM-1	CTX-M-15, TEM-1, SHV-1	IncFII	ST377	Urine	Infectious	CAZ, CTX, FEP, AMK, GEN, CIP	8	32	128
**LO-277**	*K. pneumoniae*	NDM-1	CTX-M-15, TEM-1	UT	ST2012	Cerebrospinal fluid	Infectious	CAZ, CTX, FEP, CIP	8	8	8
**LO-278**	*K. pneumoniae*	OXA-48	CTX-M-15, TEM-1, SHV-1	IncL/M	ST377	Blood	Infectious	CAZ, CTX, FEP, GEN, CIP	8	16	8
**LO-279**	*K. pneumoniae*	NDM-1	CTX-M-15, SHV-11	IncFII	ST147	Blood	Infectious	CAZ, CTX, FEP, GEN, CIP	8	32	32
**LO-4**	*E. coli*	OXA-48	CTX-M-15	IncL/M	ND	Urine	Internal of women	CAZ, CTX, FEP, CIP	1	0/125	>4
**LO-35**	*E. coli*	OXA-48	CTX-M-15	IncL/M	ST410	Urine	Poisoning ICU	CAZ, CTX, FEP, GEN, CIP	0/5	0/125	>4
**LO-96**	*E. coli*	OXA-48	CTX-M-15, TEM-1	IncL/M	ND	Wound	Infectious	CAZ, CTX, FEP, GEN, CIP	1	0/5	>4
**LO-175**	*E. coli*	OXA-48	CTX-M-15, TEM-1	IncL/M	ST1431	Urine	Emergency ICU	CAZ, CTX, FEP, CIP	2	0/5	>4
**LO-180**	*E. coli*	OXA-48	CTX-M-15, TEM-1	IncL/M	ST3134	Urine	Outpatient	CAZ, CTX, FEP, AMK, GEN, CIP	1	0/125	>4
**LO-183**	*E. coli*	OXA-48	–	IncL/M	ST5114	Urine	Outpatient	CAZ, CTX, FEP, CIP	2	0/125	>4
**LO-231**	*E. coli*	NDM-1	CTX-M-15, TEM-1	IncA/C	ST131	Urine	Internal emergency	CAZ, CTX, FEP, GEN, CIP	2	1	>4
**LO-233**	*E. coli*	OXA-48	CTX-M-15	IncL/M	ST5114	Urine	Emergency ICU	CAZ, CTX, FEP, CIP	1	0/125	>4
**LO-112**	*S. marcescens*	OXA-48	CTX-M-15, TEM-1, SHV-12	IncL/M	–	Blood	Poisoning ICU	CAZ, CTX, FEP, CIP	8	16	4
**LO-113**	*S. marcescens*	OXA-48	CTX-M-15, TEM-1, SHV-12	IncL/M	–	Blood	Poisoning ICU	CAZ, CTX, FEP, CIP	8	32	32
**LO-133**	*S. marcescens*	OXA-48	CTX-M-15, TEM-1, SHV-12	IncL/M	–	Tracheal	Poisoning ICU	CAZ, CTX, FEP, CIP	8	16	8
**LO-166**	*S. marcescens*	OXA-48	CTX-M-15, TEM-1, SHV-12	IncL/M	–	Tracheal	Poisoning ICU	CAZ, CTX, FEP, CIP	8	16	16
**LO-207**	*S. marcescens*	OXA-48	CTX-M-15, TEM-1, SHV-12	IncL/M	–	Tracheal	Poisoning ICU	CAZ, CTX, FEP, CIP	8	16	16
**LO-273**	*E. cloacae*	NDM-1	CTX-M-15, TEM-1	IncFII	ST78	Urine	Outpatient	CAZ, CTX, FEP, AMK, GEN, CIP	8	32	32
**N-20-LO**	*E. cloacae*	NDM-1	CTX-M-15, TEM-1	IncFII	ST175	Urine	General ICU	CAZ, CTX, FEP, GEN, CIP	8	4	4

*^*a*^Incompatibility (Inc) group. ND, not determined; UT, untypeable.; F, female; M, male; MIC, minimal inhibitory concentrations; S, susceptible; IPM, imipenem; MEM, meropenem; ETP, ertapenem; CAZ, ceftazidime; CTX, cefotaxime; FEP, cefepime; ATM, aztreonam; AMK, amikacin; GEN, gentamicin; CIP, ciprofloxacin; CST, colistin. Only carbapenemase producing isolates were MLST analyzed.*

### Carbapenemase and ESBL Genes

The genotyping results of carbapenemase and ESBL genes among CRE isolates are shown in Table 1. Of the 71 CRE isolates, 62 were carbapenemase producers. Among the 62 carbapenemase-producing isolates, 29 were found positive for the *bla*_NDM–1_ gene, 23 were positive for the *bla*_OXA–48_ gene, and ten of the *bla*_NDM–1_-positive isolates co-harbored *bla*_OXA–48_ genes. Among the *bla*_NDM–1_-positive *Enterobacterales* species, 26 *K. pneumoniae* isolates, two *E. cloacae* isolates, and a single *E. coli* isolate were identified. The twenty-three *bla*_OXA–48_ producers were *K. pneumoniae* (*n* = 11), *E. coli* (*n* = 7), and *S. marcescens* (*n* = 5). All the ten isolates co-producing *bla*_NDM–1_ and *bla*_OXA–48_ were *K. pneumoniae*. Other carbapenemase genes (*bla*_GES_, *bla*_KPC_ *bla*_VIM_, and *bla*_IMP_) were not detected. Among the 71 CRE isolates, 91.5% (65/71) ESBL producers were observed. Out of 65 ESBL producers, 64 (98.4%) harbored *bla*_CTX–M–15_ and eight (12.3%) harbored *bla*_SHV–12_; furthermore, a lot of isolates harbored additional *bla*_TEM/SHV_ genes.

### Clonal Relationship of CRE Isolates

**FIGURE 1:**
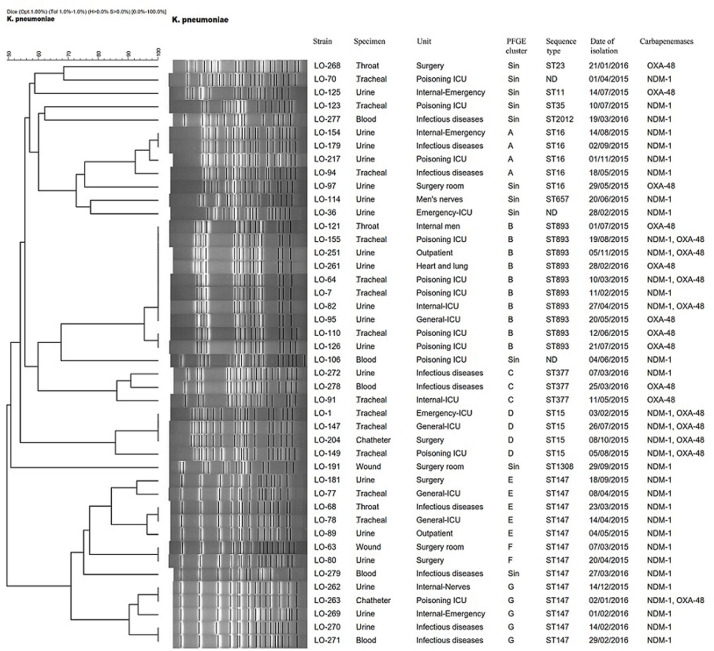
Dendrogram based on PFGE of 44 isolates of CPKP and their ST determined via MLST. ND, not determined.

**FIGURE 2:**
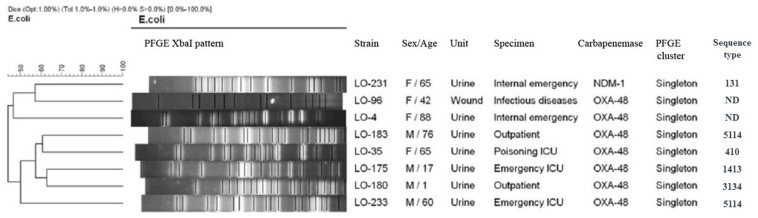
Dendrogram based on PFGE of 8 isolates of carbapenemase-producing *Escherichia coli* and their ST determined via MLST. ND, not determined.

**FIGURE 3:**
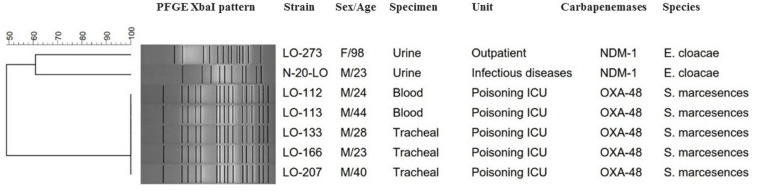
*Serratia marcescens* and *E nterobacter cloacae* are grouped together in the same dendrogram for comparison. Dendrogram based on PFGE of 5 isolates of OXA-48-producing *S. marcescens* and 2 NDM-1-producing *E. cloacae.*

Based on a cutoff of 80% genetic similarity, PFGE revealed that 44 carbapenemase-positive *K. pneumoniae* isolates could be categorized in seven clusters A (4 isolates), B (10 isolates), C (3 isolates), D (4 isolates), E (5 isolates), F (2 isolates), and G (5 isolates), while 11 isolates appeared to be singletons (Figure 1). Clusters E, F, and G belonged to ST147, while clusters A, B, C, and D were categorized as ST16, ST893, ST377, and ST15, respectively. The eight NDM-1- and OXA-48-producing *E. coli* isolates were clonally unrelated by PFGE (Figure 2), including two belonging to the same sequence type (ST5114). The PFGE patterns of five OXA-48-positive *S. marcescens* isolates showed 100% similarity, but the two NDM-1-positive *E. cloacae* had distinct PFGE patterns (Figure 3).

### Plasmid Replicon Typing and Conjugation Assay

The *bla*_NDM–1_ gene was identified on an IncFII-type plasmid for twenty-six *K. pneumoniae* and two *E. cloacae* isolates and on an IncA/C-type plasmid for a single *E. coli* isolate, while the *bla*_OXA–48_ gene was identified on an IncL/M-type plasmid for nineteen *K. pneumoniae*, seven *E. coli*, and five *S. marcescens* isolates. In the six *K. pneumoniae* isolates, we could not identify the incompatibility group.

Conjugation experiments revealed that all of the NDM-1 and OXA-48 plasmids were successfully transferred to *E. coli* K12, conferring resistance to carbapenems and cephalosporins in transconjugants. In addition, co-transfer of *bla*_NDM–1_, *bla*_OXA–48_, and other resistance determinants (*bla*_CTX–M_, *bla*_TEM_, and *bla*_SHV_) was observed in several isolates (Table 2). Plasmid gel extraction followed by PCR amplification of the transconjugants revealed that the *bla*_OXA–48_ gene was harbored on transferable plasmids belonging to the IncL/M incompatibility group, while the *bla*_NDM–1_ gene was located on conjugative plasmids. Transconjugant Tc-Lo204 had two different plasmids, and the size of one plasmid was ∼140 kb with *bla*_NDM–1_ and the other one was ∼135 kb with *bla*_OXA–48_. Notably, all *bla*_OXA–48_-positive conjugative plasmids co-harbored beta-lactamase gene *bla*_CTX–M–15_.

**TABLE 2:** Microbiological characteristics of nine clinical CPE isolates and their transconjugants.

Isolate	Species	ST	MIC (mg/L)			Antimicrobial resistance phenotype	β-lactamase(s)	Size of plasmids	Inc group
			
			ERT	MEM	IPM				
LO-35	*E. coli*	410	0.5	0/125	>4	CAZ, CTX, FEP, CIP	OXA-48, CTX-M-15	∼39 kb	IncL/M
Tc-LO-35^a^		–	0/125	0/125	>4	CAZ, CTX, FEP	OXA-48, CTX-M-15	∼39 kb	IncL/M
LO-89	*K. pneumoniae*	ST147	8	16	32	CAZ, CTX, FEP, AMK, GEN, CIP	NDM-1, CTX-M-15, TEM, SHV	104.8 ± 3.6	IncFII
Tc-LO-89^a^		–	4	8	ND	CAZ, CTX, FEP	NDM-1, CTX-M-15, TEM	–	IncFII
LO-112	*S. marcescens*	–	8	16	4	CAZ, CTX, FEP, CIP	OXA-48, CTX-M-15, TEM, SHV	∼39 kb	IncL/M
Tc-LO-112^a^		–	4	4	2	CAZ, CTX, FEP	OXA-48, CTX-M-15, TEM	∼39 kb	IncL/M
LO-149	*K. pneumoniae*	ST15	8	32	32	CAZ, CTX, FEP, AMK, GEN, CIP	NDM-1, OXA-48, CTX-M-15, TEM, SHV	130.6 ± 3.2	IncFII, IncL/M
Tc-LO-149^a^		–	8	16	8	CAZ, CTX, FEP, AMK, GEN	NDM-1, SHV	130.6 ± 3.2	IncFII
LO-155	*K. pneumoniae*	ST893	8	32	32	CAZ, CTX, FEP, CIP	NDM-1, OXA-48, CTX-M-15, TEM, SHV	–	IncL/M
Tc-LO-155^a^		–	8	8	>4	CAZ, CTX, FEP	OXA-48, CTX-M-15, TEM	–	IncL/M
LO-179	*K. pneumoniae*	ST16	8	32	24	CAZ, CTX, FEP, AMK, GEN, CIP	NDM-1, CTX-M-15, TEM, SHV	–	IncFII
Tc-LO-179^a^		–	4	4	ND	CAZ, CTX, FEP, AMK, GEN	NDM-1, TEM	–	IncFII
LO-204	*K. pneumoniae*	ST15	8	32	256	CAZ, CTX, FEP, AMK, GEN, CIP	NDM-1, OXA-48, CTX-M-15, TEM, SHV	140.2 ± 3.2 135.1 ± 3.0	IncFII, IncL/M
Tc-LO-204^a^		–	8	ND	ND	CAZ, CTX, FEP, AMK, GEN	NDM-1, OXA-48, TEM, SHV	140.2 ± 3.2 135.1 ± 3.0	IncFII, IncL/M
LO-271	*K. pneumoniae*	ST147	8	16	32	CAZ, CTX, FEP, AMK, GEN, CIP	NDM-1, CTX-M-15, TEM, SHV	107.4 ± 4.6 86.3 ± 4.8	IncFII
Tc-LO-271^a^		–	4	8	ND	CAZ, CTX, FEP	NDM-1, CTX-M-15, TEM	–	IncFII
LO-273	*E. cloacae*	ST78	8	32	24	CAZ, CTX, FEP, AMK, GEN, CIP	NDM-1, CTX-M-15	–	IncFII
Tc-LO-273^a^		–	8	ND	ND	CAZ, CTX, FEP, AMK, GEN	NDM-1	∼50 kb	IncFII

*MIC, minimal inhibitory concentrations; ERT, ertapenem, MEM, meropenem; IPM, imipenem; CAZ, ceftazidime; CTX, cefotaxime; FEP, cefepime; AMK, amikacin; GEN, gentamicin, CIP, ciprofloxacin, CST, colistin. ^*a*^Tc, *E. coli* K12 transconjugants selected in media containing 1 μg/ml MEM. Plasmid size for LO-89, LO-149, LO-204, and LO-271 isolates was estimated by ODM; that for other isolates was estimated by plasmid preparation.*

### Optical DNA Mapping

The presence of the *bla*_NDM–1_ gene on plasmids of isolates LO94, LO204, LO271, LO247, LO64, LO63, LO89, and LO149 was characterized using optical DNA mapping (ODM). Table 3 presents a summary of the ODM data for the *bla*_NDM–1_-carrying plasmids in these eight *K. pneumoniae* strains. DNA barcodes for each isolate were clustered based on similarity, and clusters with consensus cut sites (with at least nine barcodes) were used to infer the Cas9 cutting, suggesting the presence of the *bla*_NDM–1_ gene on the plasmids (Müller and Westerlund, 2017). For isolate LO271, two plasmids (∼86 kb and ∼107 kb) carrying the *bla*_NDM–1_ gene were identified. For isolate LO204, two plasmids of length ∼140 kb and ∼135 kb were found; however, only the ∼140-kb plasmid carried the *bla*_NDM–1_ gene. The remaining six isolates carried only one plasmid in the size range ∼110 kb to ∼130 kb carrying the *bla*_NDM–1_ gene.

**TABLE 3:** Clinical and ODM information about *bla*_NDM–1_-carrying plasmids in eight *K. pneumoniae* strains isolated from Loghman hospital in Tehran.

Strain no.	MIC (μ g/ml)			Species	ST	*bla*_NDM–1_ carrying plasmids (kbp)	
	
	ERT	MEM	IPM				
LO-94	8	4	4	*K. pneumoniae*	16	126.3 + 5.1	
LO-204	8	32	256	*K. pneumoniae*	15	140.2 ± 3.2	135.1 ± 3.0*
LO-271	8	16	32	*K. pneumoniae*	147	86.3 ± 4.8	107.4 ± 4.6
LO-247*	8	8	4	*K. pneumoniae*	ND*	110.7 ± 2.5	
LO-64	8	32	48	*K. pneumoniae*	893	125.4 ± 3.0	
LO-63	8	32	32	*K. pneumoniae*	147	122.7 ± 2.9	
LO-89	8	16	32	*K. pneumoniae*	147	104.8 ± 3.6	
LO-149	8	32	32	*K. pneumoniae*	15	130.6 ± 3.2	

*Did not carry *bla*_NDM–1_. ND, not determined.

**FIGURE 4:**
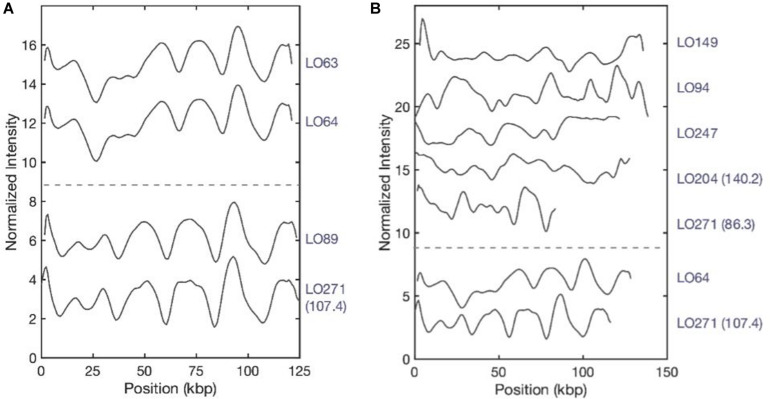
Plasmid barcodes of *bla*_NDM–1_-carrying plasmids in eight *Klebsiella pneumoniae* strains. Since the plasmids are linearized by Cas9-targeting *bla*_NDM–1_, all barcode ends are where we locate the *bla*_NDM–1_ gene. For the samples containing two *bla*_NDM–1_ plasmids, sizes are written in brackets to differentiate the plasmids. **(A)** Identical plasmids with the same sizes (∼125 kb) and the same location of *bla*_NDM–1_. **(B)** Plasmids encoding *bla*_NDM–1_ that do not match among each other. LO-271 (107.4) and LO-64 were plotted here for reference.

After plasmid size estimation and *bla*_NDM–1_ gene detection, we compared the consensus barcodes among the eight isolates (Figure 4). The ODM assay showed that identical plasmids with the same size (∼125 kb) and the same location of the *bla*_NDM–1_ were found in LO63 and LO64 (Figure 4A). These isolates belong to sequence types ST147 and ST893, respectively (Figure 1), suggesting a possible transmission of plasmid from one strain to the other. Similarly structured plasmids were found in LO89 and LO271 (∼107 kb) (Figure 4A); they both belong to the same sequence type, ST147. By visual inspection, it appears that large regions of the plasmids of isolates LO63, LO64, LO89, and LO271 (Figure 4A) are similar, further accentuated by the fact that the *bla*_NDM–1_ gene is located at the same position. There are however, other regions that are not the same, and the size differs (plasmids from LO63, LO64, and LO89 were ∼125 kb while the plasmid from LO271 was ∼107 kb). The plasmids from the other isolates do not match among each other (Figure 4B) or with the plasmids in Figure 4A. In total, we therefore found seven different plasmids carrying the *bla*_NDM–1_ gene.

## Discussion

Herein, we found 71 CRE in a period of 1 year with a lot of CPE species from patients in the same hospital in Tehran, Iran, and major dissemination of the *bla*_NDM–1_ and *bla*_OXA–48_ genes, which might be considered endemic in the geographical area, through the spread of conjugative plasmids.

The co-occurrence of NDM-1- and OXA-48-producing *Enterobacterales* species is also considerable since the identification of NDM-1 and OXA-48 producers in Iran (Solgi et al., 2017b), Lebanon (Dandachi et al., 2016), and Kuwait (Jamal et al., 2015) shows that these carbapenemases, known to be widespread in the Indian subcontinent, may also be widespread in the Middle East. In our study, the majority of the NDM-1- and OXA-48-producing *Enterobacterales* isolates co-harbored at least one ESBL gene which is concordant with previous reports (Torres-González et al., 2015; Solgi et al., 2017a). In this study, nine carbapenem-resistant *K. pneumoniae* were identified; this may be due to other resistance mechanisms (e.g., more rare carbapenemases, porin loss, AmpC enzymes) that were not investigated in detail in this study.

The plasmid incompatibility types IncFII and IncA/C were identified among the NDM-1-producing isolates, while only IncL/M was detected among OXA-48 producers (Table 1). These replicon types have been reported in *Enterobacterales* species in many regions of the world (Brañas et al., 2015; Guo et al., 2016; Kieffer et al., 2016; Solgi et al., 2017b). Also, Weber et al. (2019) demonstrated that the potential transmission of mobilized Tn125-like transposons with *bla*_NDM–1_ into different plasmids among *Enterobacterales* species (Weber et al., 2019). Conjugation assays were successful for all CPE isolates and allowed the identification of *bla*_OXA–48_-carrying plasmids belonging to the IncL/M incompatibility group in all transconjugants, with the exception of Tc-LO-149 (Table 2). Also, analysis of transconjugants showed that the *bla*_NDM–1_ carried on transferable plasmids belonging to the IncFII and IncA/C incompatibility group, respectively.

The identification of conjugative plasmids harboring *bla*_NDM–1_ and *bla*_OXA–48_ genes in CRE isolates shows that these plasmids contribute to the dissemination of carbapenemase genes among *Enterobacterales* species. Therefore, resistance to carbapenems in CRE isolates is likely to be associated with the spread of these genes in this hospital, which is consistent with previous studies (Jamal et al., 2016; Kieffer et al., 2016; Solgi et al., 2017a).

Pulsed-field gel electrophoresis revealed that different clones of carbapenemase-producing *K. pneumoniae* (CPKP) were present, and there were two predominated clones that were identified as ST147 and ST893, comprising 13 and 10 isolates, respectively. ST147 and ST893 have been circulating in this hospital setting during the period of investigation, indicating two separate outbreaks, with the ICU poisoning acting as the epicenter. Indeed, hospital outbreaks of ST147 NDM-1-producing *K. pneumoniae* are common in Europe (Bogaerts et al., 2011; Giske et al., 2012), whereas the outbreak of OXA-48-producing ST893 *K. pneumoniae* was only reported from Isfahan, Iran (Solgi et al., 2018).

The dominant endemic sequence type *K. pneumoniae* in our study was ST147 which co*-*harbored NDM-1 and *bla*_CTX–M–15_, *bla*_TEM–1_, and *bla*_SHV–11,12,172_ genes. As an internationally successful sequence type, ST147 has previously been linked to the spread of ESBLs (especially CTX-M-15), OXA-48, VIM, and KPC and recently also to NDM-1 in various countries (Bogaerts et al., 2011; Messaoudi et al., 2017). In addition, ST893, the second most common sequence type in this study that co-harbored *bla*_CTX–M–15_, *bla*_TEM–1_, and *bla*_SHV–199_, has also only been reported in Iran among CPKP isolates which has been associated with ESBL and carbapenemase genes (Solgi et al., 2018). Several other STs were found among CPKP isolates, including ST16 (cluster A), ST377 (cluster C), ST15 (cluster D), ST11, ST23, ST35, ST2012, ST657, and ST1308.

The four isolates of ST15 (cluster D) were isolated from patients in four ward. All isolates carried *bla*_NDM–1_ in combination of *bla*_OXA–48_ and *bla*_CTX–M–15_, *bla*_TEM–1_, and *bla*_SHV–106_ genes. *K. pneumoniae* ST15 represents a single locus variant of ST14 and is currently widely disseminated among CTX-M-15- and OXA-48- or NDM-1-producing *K. pneumoniae* isolates in different geographical regions (Poirel et al., 2014; Kieffer et al., 2016; Ben et al., 2017; Jelić et al., 2017).

The four NDM-1- and one OXA-48-producing *K. pneumoniae* in our study belonged to ST16 and were positive for *bla*_CTX–M–15_ and *bla*_SHV–199_ genes. It is noteworthy that OXA-48-producing ST16 have also been described in *K. pneumoniae* that caused outbreaks in two hospitals in different regions of Spain (Oteo et al., 2013). Furthermore, two OXA-48- and one NDM-1-producing *K. pneumoniae* were isolated from three patients in two different ward. They belonged to ST377, which has previously not been reported as a carbapenemase producer. Finally, one OXA-48-producing *K. pneumoniae* isolate that co-carried *bla*_CTX–M–15_, *bla*_TEM–1_, and *bla*_SHV–182_ was identified as ST11. The *bla*_OXA–48_-harboring IncL/M plasmids have been mainly described in *K. pneumoniae* ST11 in different countries including, Spain (Brañas et al., 2015), Taiwan (Ma et al., 2015), and Greece (Voulgari et al., 2013).

Considering this study and our previous study in Isfahan province (Solgi et al., 2018), the main *K. pneumoniae* STs that were identified in Iran were ST893, ST11, and ST147. This scenario suggests that these STs have likely been circulating in Iran in recent years. Our results show that, in general, the population structure of CP *E. coli* is more diverse than that of CPKP, which is essentially similar to the findings of other studies (Sartor et al., 2014; Kieffer et al., 2016; Solgi et al., 2017b). We detected *E. coli* ST410, ST1431, ST3134, and ST5114 which have been reported as harboring *bla*_OXA–48_ and ESBL genes. Moreover, we identified only one ST131 of *E. coli* which harbored *bla*_NDM–1_, *bla*_CTX–M–15_, and *bla*_TEM–1_ genes. The association of NDM-1 and ESBL genes with the pandemic clone ST131 has been previously reported from several countries (Peirano et al., 2011, 2014).

The two NDM-1-positive *E. cloacae* isolates were genetically not related and belonged to two STs, ST78 and ST175, both also carried *bla*_CTX–M–15_ and *bla*_TEM–1_ genes, while the five *S. marcescens* isolates were considered identical (>99% similarity). Interestingly, looking at the hospitalization ward from which the patients originated, several infections were detected at the ICU poisoning, with a total of five patients harboring this OXA-48-producing *S. marcescens* strain which co-carried further beta-lactamase genes (*bla*_CTX–M–15_, *bla*_SHV–12_, and *bla*_TEM–1_). Our results showed that this OXA-48-producing *S. marcescens* strain was isolated among inpatients who shared a room. Therefore, it is possible that the spread of this strain from patient to patient occurred. To the best of our knowledge, this is the first report of an outbreak of OXA-48-producing *S. marcescens* that co-harbored ESBL genes in Iran. A small hospital outbreak linked to OXA-48-producing *S. marcescens* has been previously reported in Lebanon (Hammoudi et al., 2014). The exact mechanism of CPE spread in Iran is not well understood. Our previous study in July to November 2015 in two university hospitals in Iran showed that the rate of fecal carriage of CRE among inpatients is high (37.9%) and predominant species were *K. pneumoniae*, *E. coli*, *E. cloacae*, and *Proteus mirabilis*, which harbored the *bla*_NDM–1_ and *bla*_OXA–48_ genes (Solgi et al., 2017a). The circulation of *bla*_NDM–1_ and *bla*_OXA–48_ carbapenemase genes in the general population may result in a further spread by traveling and continuous introduction into the hospitals.

In conclusion, findings of extensive analysis of plasmids in the present study showed the enormous potential of spread of carbapenemase genes by horizontal gene transfer via plasmids and we identified the conjugative plasmids carrying the *bla*_NDM–1_ and *bla*_OXA–48_ genes in different *Enterobacterales* species that co-produce ESBLs. Here, in one Tehran hospital, we report two separate outbreaks of NDM-1-producing ST147 and OXA-48-producing ST893 *K. pneumoniae* STs. Furthermore, an outbreak with OXA-48-producing *S. marcescens* was observed. It is necessary to continue epidemiological and active surveillance to improve the control and prevention of infections associated with CPE isolates in healthcare facilities.

## Data Availability Statement

The raw data supporting the conclusion of this article will be made available by the authors, without undue reservation, to any qualified researcher.

## Ethics Statement

The study was approved by the research and the Ethics Committee of the Pasteur Institute of Iran (No. 1395.51). No ethical approval was obtained for using the clinical isolates since they were collected during the routine diagnostic laboratory at our hospital.

## Author Contributions

HS and FS designed the study. HS, SN, Y-LL, GG, VN, and AN carried out the experiments. HS, CG, FB, FW, and FS analyzed the data. HS, CG, FW, and FS wrote the manuscript.

## Conflict of Interest

The authors declare that the research was conducted in the absence of any commercial or financial relationships that could be construed as a potential conflict of interest.

## Footnotes

1 https://bigsdb.pasteur.fr/klebsiella/klebsiella.html

2 https://enterobase.warwick.ac.uk/species/ecoli/allele_st_search

3 https://pubmlst.org/ecloacae/
